# Hepatic Disease the Cause of Hypochondriasis

**Published:** 1811-09

**Authors:** 


### *Hepatic Disease the Cause of Hypochondriasis.*

(Month. Mag.)

It has been considered that the only bodily disease, under which the hypochondriacal patient appeared to labour, consisted in a disordered state of the stomach. That the stomach is generally disordered in such cases is certain, indeed it seems probable that no one of the digestive organs could be materially disordered without the others participating; but it is also probable, when hypochondriasis occurs, that the liver is the viscus, whose functions are principally deranged. Before the publication of Mr. Abernethy's excellent

" Observations

"Observations on the Constitutional Origin and Treatment of Local Diseases," it was thought that there must be some striking peculiarity in the disorder of the digestive viscera, when those remarkable and distressing feelings occurred which have been called hypochondriasis and melancholia; but it never occurred till that work appeared, that such peculiarity might consist in a derangement of the hepatic functions in particular. This seems an important thing to know, because many of those medicines which in other cases would strengthen and evacuate the stomach and bowels, would not restore those organs to a healthy state, while the liver remained the principal seat of the disorder, which might subside after the administration of small doses of *Pil. Hydrarg.** Mr. Abernethy justly reminds his readers, that the terms used by the ancients to express a dejected and irrational state of mind, had all a reference to hepatic disorder. Melancholia from μηλας and χολη, hypochondriasis from ὑπο and χονδριον, as well as the terms *atrabilius* and *manie atrabiliaire*, all signify disorder of the liver.† Indeed the subsisting connection between the state of this organ and that of the mind, was so generally known to the antients, that it was frequently alluded to by their poets, and metaphorical allusions to hepatic disorders were made use of to express mental perturbation. Thus Horace,

> "Cum tu, Lydia, Telephi
> Cervicem roseam, cerea Telephi
> Laudas brachia, væ, meum
> Fervens difficili bile *tumet jecur.*
> Tunc nec mens mihi, &c."—*Lib.* 1. *Carm.* xiii.

And again,

> Quum tibi flagrans amor, et libido,
> Quæ solet matres furiare equorum,
> Sæviet circa *jecur ulcerosum,*
> Non sine questu. *Lib.* 1, *Carm.* xxv.

And Juvenal,

> "Quid referam? quantâ siccum jecur ardeat irâ,

> Cum populum gregibus comitum premit hic spoliator, &c. *Sat.* i, 45.

Again,

> "Rumpe miser *tensum jecur.*"

---

* See Surgical Observations on the Constitutional Origin and Treatment of Local Diseases, &c. p. 213.

† Some modern writers have absurdly called this disorder the *spleen*, while others, influenced by the whimsical humoral pathology, have denominated it the *vapours*.

Perseus

Perseus says,

———" *rupto jecore* exierit caprificus ?

En pallor, seniumque. O mores," &c. *Satyr.* i. 26.

The following passage is still more to the purpose :

———" nec quicquam extrinsecus intrat,

Quod nervos agitet ; sed si intus et in *jecore ægro*

Nascantur," &c. *Satyr.* v. 129.

Ovid, unable to account for a bodily infirmity under which he laboured, and, supposing he could not be bewitched, exclaims,

" Sagave puniceâ defixit nomina cerâ,
Et medium tenues in *jecur* egit acus." *Lib. amor.*

See also some extraordinary assertions about the liver in *Plin. Hist. Nat. lib.* xi. *cap.* 37.

